# The impact of the HIV epidemic on U.S. anal cancer rates, 1980-2007

**DOI:** 10.1186/1750-9378-7-S1-O10

**Published:** 2012-04-19

**Authors:** Meredith S Shiels, Ruth M Pfeiffer, Aimée R Kreimer, Anil K Chaturvedi, Eric A Engels

**Affiliations:** 1 Division of Cancer Epidemiology and Genetics, National Cancer Institute, National Institutes of Health, Bethesda, MD, USA

## Background

U.S. incidence rates of anal cancer have increased steadily over time, and are generally higher in women than men. It has been proposed that the HIV epidemic may have influenced U.S. anal cancer trends. Compared to the general population, anal cancer rates are strongly elevated in HIV-infected individuals. Anal cancer rates in HIV-infected individuals have also increased over time. We estimated the impact of the HIV epidemic on U.S. anal cancer trends during 1980-2007.

## Methods

Data on anal cancer incidence rates were obtained from the HIV/AIDS Cancer Match Study, which links 14 U.S. HIV/AIDS and cancer registries. We estimated incidence rates with anal cancer cases and person-years for the general population, and then subtracted 2 groups of cases in people with AIDS: cases that occurred after AIDS diagnosis (incident) and within 5 years prior to AIDS (prevalent). All rates were standardized to the 2000 U.S. population by age, sex and race. Poisson regression was used to estimate changes in rates over time.

## Results

**Figure 1:**
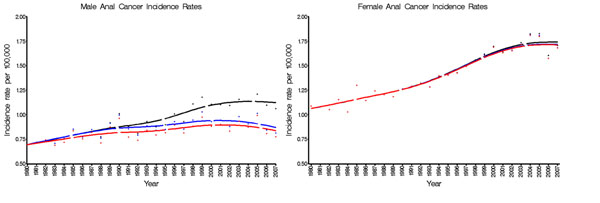
Male (A) and female (B) anal cancer incidence rates in overall (black line), excluding incident cases in people with AIDS (blue line), and excluding incident and prevalent cases in people with AIDS (red line), 1980-2007.

During 1980-2007, a total of 25,011 anal cancers occurred in 2.1 billion person-years of follow-up. Of these, 1087 were incident and 456 were prevalent cases in people with AIDS. Among men, the anal cancer rate increased 2.0% per year from 0.69 to 1.06/100,000 during 1980-2007 (Figure 1A). Excluding cases in people with AIDS, the rate only increased 0.77% per year to 0.77/100,000 in 2007. Among women, the anal cancer rate increased 2.1% per year from 1.09 to 1.71/100,000 during 1980-2007 (Figure 1B). Removal of cases with AIDS changed the trends very little (increase of 2.1% per year). Among 20-49 year olds, AIDS cases strongly influenced trends in men. Overall rates increased 4.0% per year, but rates excluding AIDS cases increased only 0.72% per year. In contrast, among women aged 20-49 years, AIDS had little impact on annual anal cancer rates (3.7% overall vs. 3.4% excluding AIDS cases). In 70+ year olds, the age group with the highest anal cancer incidence, AIDS had a small effect on male and no effect on female trends. During 2003-2007, 24.2% of anal cancers among men and 1.6% among women occurred in people with AIDS.

## Conclusions

During 1980-2007, the U.S. anal cancer epidemic in young men was strongly influenced by the HIV epidemic; however, among women, the anal cancer epidemic was independent of HIV. Effective anal cancer prevention in HIV-infected men would have a substantial impact on U.S. anal cancer rates.

